# Evaluation of a Model-Based Hemodynamic Monitoring Method in a Porcine Study of Septic Shock

**DOI:** 10.1155/2013/505417

**Published:** 2013-03-25

**Authors:** James A. Revie, David Stevenson, J. Geoffrey Chase, Chris J. Pretty, Bernard C. Lambermont, Alexandre Ghuysen, Philippe Kolh, Geoffrey M. Shaw, Thomas Desaive

**Affiliations:** ^1^Department of Mechanical Engineering, Centre of Bioengineering, University of Canterbury, Private Bag 4800, Christchurch 8140, New Zealand; ^2^Hemodynamic Research Laboratory, University of Liege, Liege, Belgium; ^3^Department of Intensive Care, Christchurch Hospital, Christchurch, New Zealand

## Abstract

*Introduction*. The accuracy and clinical applicability of an improved model-based system for tracking hemodynamic changes is assessed in an animal study on septic shock. *Methods*. This study used cardiovascular measurements recorded during a porcine trial studying the efficacy of large-pore hemofiltration for treating septic shock. Four Pietrain pigs were instrumented and induced with septic shock. A subset of the measured data, representing clinically available measurements, was used to identify subject-specific cardiovascular models. These models were then validated against the remaining measurements. *Results*. The system accurately matched independent measures of left and right ventricle end diastolic volumes and maximum left and right ventricular pressures to percentage errors less than 20% (except for the 95th percentile error in maximum right ventricular pressure) and all *R* ^2^ > 0.76. An average decrease of 42% in systemic resistance, a main cardiovascular consequence of septic shock, was observed 120 minutes after the infusion of the endotoxin, consistent with experimentally measured trends. Moreover, modelled temporal trends in right ventricular end systolic elastance and afterload tracked changes in corresponding experimentally derived metrics. *Conclusions*. These results demonstrate that this model-based method can monitor disease-dependent changes in preload, afterload, and contractility in porcine study of septic shock.

## 1. Introduction

Cardiovascular management of the critically ill is often difficult due to the array of complex circulatory interactions that occur within hemodynamically compromised patients. The limited measurement set typically available in the intensive care unit (ICU) is sometimes insufficient to give a full picture of cardiovascular status. Hence, diagnosis and therapy are often based on the experience, skill, and intuition of the attending medical staff. However, difficulties in interpreting cardiac and circulatory measurements can result in misdiagnosis and suboptimal treatment, which may lead to inefficient use of resources, increased length of stay, and in some cases death [1–5].

To improve hemodynamic monitoring, model-based methods can be used to aggregate common ICU measurements into an easily understandable, physiological form. Other model-based methods have been used to estimate surrogate markers of cardiovascular performance from clinically available data. However, these approaches have only been used to identify a small number of hemodynamic parameters [6] or only describe localised behaviour [7, 8], normally to obtain estimates of CO [9–16]. To truly understand the effects of many common cardiovascular diseases in the ICU, one must consider the global effects these diseases have on a patient. Hence, a broader approach that describes the effects of disease on parameters of cardiac function, as well as parameters of systemic and pulmonary flows, is required. Patient-specific models, identified from clinical data, could be used to help paint a clearer physiological picture of the patient's global cardiovascular condition and assist medical staff with decision making [17, 18]. The goal of this research is to show that such an *in silico* system can accurately identify important disease and treatment dependent changes in the cardiovascular system (CVS).

A previously reported model-based method [19, 20] indicated that subject-specific disease induced hemodynamic changes due to pulmonary embolism could be tracked from common cardiovascular measurements. However, the method lacked clinical applicability because invasive measurements of left and right ventricular volumes waveforms were required, which are rarely available in the ICU. Thus, an enhanced method [21, 22] has been developed that only uses clinically available or easily inferred cardiovascular measurements, so the system can be used to monitor hemodynamics in a normal, clinical setting. 

In the ICU, some of the most prevalent and deadly cardiovascular disorders are severe sepsis and septic shock [1, 2] which are caused by a whole body inflammatory response to an infection, resulting in systemic vasodilation. To further prove the monitoring ability of this new method, subject-specific CVS models are retrospectively identified and validated using measurements from a porcine study on septic shock and large-pore hemofiltration therapy [23]. Hence, in this study, the ability of subject-specific CVS models to monitor the signs of sepsis, including a decrease in systemic vascular resistance (*R*_sys_) [24–26], an increase in pulmonary vascular resistance (*R*_pul_) [26], and an increase right ventricle end diastolic elastances (RVEDV) [26], were analysed.

## 2. Materials and Methods

This study used cardiovascular measurements recorded during a porcine trial studying the efficacy of large-pore hemofiltration (LPHF) for treating septic shock [23]. Although the efficacy of LPHF is not of particular interest to this study, the induction of sepsis followed by LPHF provides a dynamic and clinically realistic background on which to test this model and methodology. Measurements taken from four pigs during this study provide a suitable dataset for identifying subject-specific CVS model parameters and safely validating the model-outputs. In this work, subject-specific CVS models were only identified from measurements commonly available in the ICU. Changes in the identified parameters in these models were analysed to track the effect of septic shock in the pigs. No prior assumptions were made or implemented regarding the trends in these identified parameters. The more invasive measurements recorded in the porcine study, which are not widely obtainable clinically, were used to validate the accuracy of the CVS models.

### 2.1. Subjects

Four pure Pietrain pigs weighing 20–30 kg were premedicated, anesthetized, and ventilated as described in [23, 27]. The animals received a 0.5-mg/kg endotoxin infusion (lipopolysaccharide from *Escherichia coli* serotype 0127:B8; Sigma Chemical, St. Louis, MO, U.S.A) over 30 minutes (from T0 to T30) to initiate sepsis. From T60 onwards the pigs underwent a zero balance continuous venovenous hemofiltration at a rate of 45 mL/kg/h with a 0.7 m^2^ large-pore (78 Å) membrane with a cut off of 80 kDa (Sureflux FH 70, Nipro, Osaka, Japan) and a BaxterBM 25-BM 14 hemofiltration device (Baxter Health Care, Munich, Germany). Each animal acted as its own control in this study with baseline measurements taken prior to the endotoxin infusions (T0), representing the undiseased state of the pig. All procedures and protocols used in the porcine experiments were reviewed and approved by the Ethics Committee of the Medical Faculty at the University of Liege (Belgium).

### 2.2. Measurements

The pulmonary trunk was accessed via medial sternotomy, and a micromanometer-tipped catheter (Sentron, Cordis, Miami, FL) was inserted into the main pulmonary artery through a stab wound in the RV outflow tract and positioned approximately 2 cm downstream of the pulmonary valve. Aortic pressure was measured using a micromanometer-tipped catheter inserted into the descending thoracic aorta through the right femoral artery. A 7F, 12 electrode conductance micromanometer-tipped catheter (CD Leycom, Zoetermeer, Holland), was positioned in each of the left and right ventricles, so that all electrodes were in their respective cavities. Further details on the trials can be found in [23].

These catheters provided measurements of the aortic and pulmonary pressure waveforms (*P*_ao_, *P*_pa_) and the left and right ventricular pressure and volume waveforms (*P*_lv_, *V*_lv_, *P*_rv_, and *V*_rv_). Continuous waveforms of *P*_ao_, *P*_pa_, *P*_lv_, *V*_lv_, *P*_rv_, and *V*_rv_ (sampling frequency, *f*_*s*_ = 200 Hz) were recorded from the four catheters at 30 minutes intervals (T0, T30, T60,…, T210, and T240) between initiation of anaesthesia (T0) and 4 hours subsequent (T240). At each 30 minute interval 6–12 heartbeats of these waveforms were recorded. For each set of waveforms one good heartbeat worth of data was selected. From this selected heartbeat discrete convergence set points for the parameter identification process were extracted. In this study, 34 sets of measurements were extracted from the raw data providing 34 sets of convergence set points from the four pigs. Two data sets were unusable as steady-state measurements could not be obtained.

### 2.3. Cardiovascular System Model

This study uses a previously validated cardiovascular system model [19, 28, 30–32] defined by a set of equations describing cardiac and circulatory flow. This model describes the “lumped” or global dynamics of major regions of the CVS, ignoring smaller localised behaviour. The lumped nature allows the model to be relatively light computationally, requiring a smaller number of parameters. However, it is complex enough to capture all the clinically important hemodynamics of the CVS.

**Figure 1:**
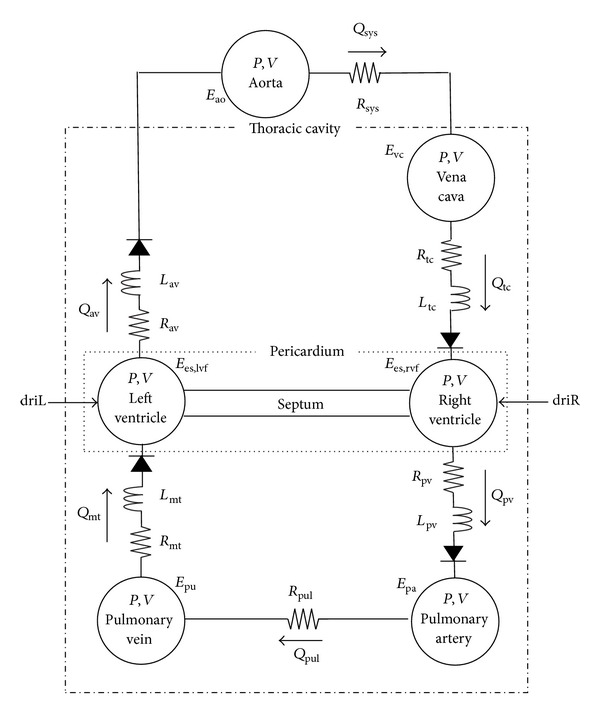
Overview of the six-chamber cardiovascular system model which relates the pressures (*P*), volumes (*V*), and flows (*Q*) in the cardiovascular system using parameters of elastance (*E*), resistance (*R*), and inertance (*L*); driL and driR represent cardiac muscle activation and act as the driver functions for the CVS model.

The six chamber CVS model represents the left and right ventricles, aorta, pulmonary artery, vena cava, and pulmonary vein, as illustrated schematically in Figure 1. Pressures and volumes in the chambers (*P*, *V*) and flows between the chambers (*Q*) are defined using parameters of resistance to flow (*R*), blood inertia (*L*), and chamber elastance or stiffness (*E*). Cardiac muscle activation of the ventricles is represented using time varying elastances (driL and driR) which act as the driver functions for the CVS model. The ventricular chambers in the model are actively elastic meaning pressure changes nonlinearly with respect to volume, to model the effects of contraction and relaxation in these chambers. The noncardiac chambers are passively elastic. Thus, pressure is linearly related to volume in these chambers. Flow in and out of the ventricles is controlled via pressure-gated heart valves, and the parallel interaction between the ventricles is modelled through pericardial and septal dynamics. The combination of all these model features and parameters provides a means for accurately depicting ventricular, arterial, and venous blood pressures and volumes, and the blood flow rates through the heart valves, body, and lungs. The full model definition can be found in Hann et al. [33].

Please note that this model does not implicitly simulate any autonomic control mechanism. However, such mechanisms can be indirectly simulated through alteration of the model parameters. For example, sympathetic control of the blood pressure could be simulated by increasing the parameter *R*_sys_ in the CVS model.

### 2.4. Parameter Identification Method: Pig-Specific Model

The parameter identification method used for this study was described by Revie et al. [21, 22]. The advantage of this method over the previously employed technique [19, 20] is that it only requires data from measurements typically available in an ICU environment.  Table 1 shows the small set of measurements necessary to identify the patient-specific parameters required for the model.

**Figure 2:**
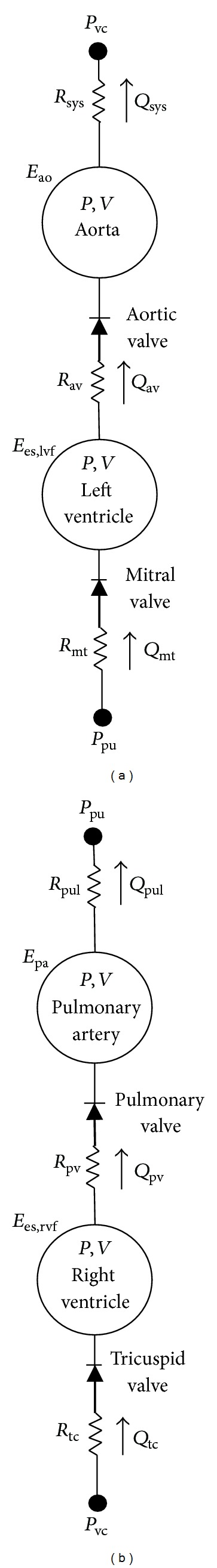
Simplified submodels of (a) the systemic and (b) pulmonary circulations with inertia and ventricular interaction removed. Note that for comparison the orientation of the pulmonary circulation has been reversed with respect to Figure 1.

The parameter identification process was performed in sequential stages to increase convergence stability. In this process the inertances in the CVS model were ignored (*L*_mt_ = *L*_av_ = *L*_tc_ = *L*_pv_ = 0), as they were found to have negligible effect on the modelled flow. To simplify parameter identification, the six-chamber CVS model was divided into two submodels of the systemic and pulmonary circulations, as shown in Figure 2. These models were decoupled by removing the ventricular interaction between the circulations. The submodels were independently simulated using left and right ventricular driver functions (driL and driR) and initial estimates for parameter values. The driver functions were identified using the method described in [34, 35] and help define the ventricular systolic and diastolic function in the identified models. These driver functions play an important role in defining pig and time dependent changes in diastolic ventricular function in the subject-specific CVS models, as the parameters of ventricular filling (as shown in ((A.4)) in the appendix) are held as constants (see Table 5) during the identification process.

An iterative proportional gain control method [22] was used to match the model outputs of Table 1 to the corresponding measurements, identifying the desired parameter set of Table 1. Each observable model output was used to identify one model parameter. Parameters (*P*) that were proportionally related to their corresponding model output were iteratively identified using the ratio of the true measurement to corresponding model output:

(1) $$\begin{array}{c} P_{\text{new}}=\frac{\text{Measurement}}{\text{Model}\;\;\text{Output}}P_{\text{old}}. \end{array}$$

Parameters that were inversely related to their chosen model output were identified using

(2) $$\begin{array}{c} P_{\text{new}}=\frac{\text{Model}\;\;\text{Output}}{\text{Measurement}}P_{\text{old}}. \end{array}$$

**Figure 3:**
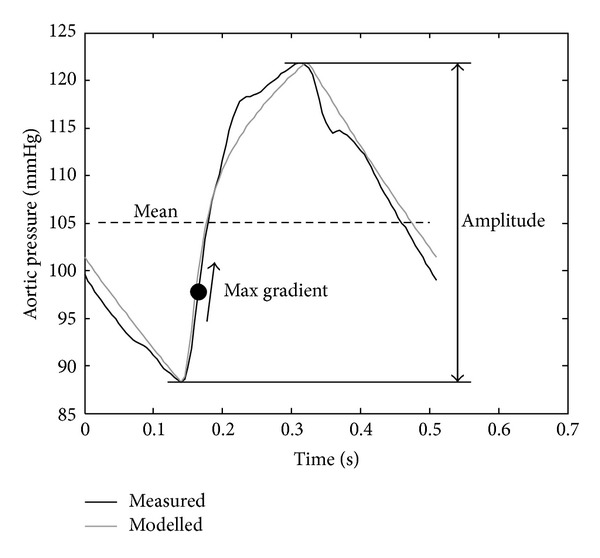
Example of the features from the aortic pressure waveform which are matched during the model identification process.

The parameters of the systemic submodels were identified first, followed by the pulmonary model. Once the both submodels were identified, they were joined together, with coupling between the models added in the form of septum and pericardium dynamics. The systemic and pulmonary models were then reidentified with the knowledge of this coupling, and the whole process was repeated until whole CVS model had converged. This process was repeated for each measurement set for that pig (i.e., T0, T30, T60,…, T240). From these identified models the valve resistances were averaged, and the remaining parameters were reidentified using these averaged valve resistances each measurement set from T0 to T240. Hence, for a given pig, nine unique sets of parameters were identified representing the hemodynamic state of the pig at the point in the experiment. A full description of the model identification process is provided by Revie et al. [22] and in the appendix for the interested reader.  Figure 3 shows an example of the systemic model matching the mean, amplitude, and maximum ascending gradient of the aortic pressure waveform, through the identification of *R*_sys_, *E*_ao_, and *R*_av_, respectively (see ((A.21)), ((A.22)), and ((A.24)) in the appendix). It should be noted that the parameter identification method reported in [22] has been modified, so the dead space of volumes of the ventricles is assumed to be equal to 23 mL, as reported in [36], rather than 0 mL.

In this study, global end diastolic volume (GEDV) was assumed to be equal to the sum of the left and right ventricular end diastolic volumes (LVEDV + RVEDV). Stroke volume (SV) was calculated as the average of the amplitudes of the left and right ventricular volume waveforms. The mitral and tricuspid valve closure times (*t*_mt_, *t*_tc_) were estimated from the derived subject-specific left and right ventricular driver function, (time varying elastances). These driver functions were calculated using the method described by Hann et al. [37] from SV and features in the aortic and pulmonary artery pressure waveforms. 

In this study, GEDV and SV were not directly measured but instead were inferred from the experimental measurements of *V*_lv_ and *V*_rv_. However, clinically, GEDV and SV can be measured using minimally invasive thermodilution techniques [38–41]. The aortic pressure waveform can be estimated from radial artery pressure using one of the several transfer function methods [42–47]. Pulmonary artery indices can be determined using a pulmonary artery catheter (PAC). Vena cava pressure, *P*_vc_, can be identified from central venous pressure which is normally measured in the ICU, removing the need to measure *t*_tc_. Mitral valve closure time, *t*_mt_, can be estimated from ECG data. Hence, the data set required for model identification represents only measurements that are already measured or inferred in a typical ICU.

### 2.5. Analysis

Data sets were recorded every 30 minutes from T0 to T240 during the four trials. However, two sets of measurements for Pig 1 at T30 and T90 were unusable, as the pig's hemodynamics were unstable at these times. Thus, 34 data sets were available for analysis. For each data set, a subject-specific CVS model was retrospectively identified from a subset of available measurements. The model identification method was run on a 2.13 GHz dual core machine with 3 GB of ram. Since this research is still in the development stages it has only been fully tested using the development-orientated but relatively slow MATLAB software (MathWorks, Natwick, MA, USA). Using one processor the identification method took on average 6 minutes and 24 seconds to identify a subject-specific model of the CVS. Preliminary tests using C programming language, which is better suited for real time applications, have suggested that the identification process time can be reduced by a factor of 20–100, to approximately 4–19 seconds per identified model, which is an acceptable run time in a clinical environment.

During parameter identification, all the parameters listed in Table 1 were uniquely identified for each set of measurements (at T0, T30,…, T240), except for the valve resistances which were averaged over the duration of the trials (T0–T240) for each pig. The model outputs of the identified models were compared to the remaining recorded measurements to validate the accuracy of the identified models. Averaged data is presented as mean ± one standard deviation (1SD). A paired-sample *t*-test was used to check temporal variance over T0–T60 to analyse the effect of the endotoxin intervention. *P* < 0.05 was considered a statistically significant result. The relationship between the modelled and measured maximum left and right ventricular pressures and volumes was analysed statistically using correlation and linear regression analysis, including calculation of absolute percentage errors, and bias and precision analysis (Bland and Altman [48]). A percentage error less than 20% was regarded as an acceptable result as measurement of physiological variables often lack precision, with errors of ±10–20% not uncommon [49–51].

## 3. Results

**Figure 4:**
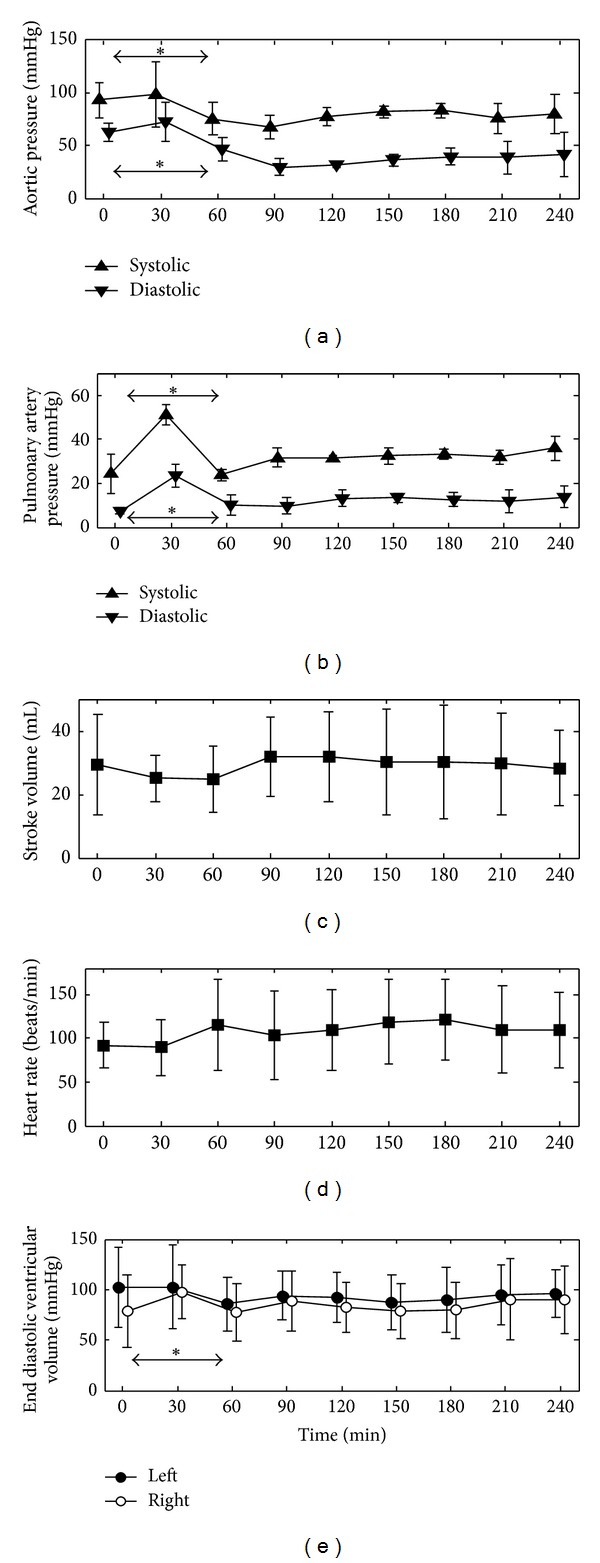
Evolution of the averaged hemodynamic measurements recorded during the trials. *indicates *P* < 0.05 for expected temporal changes over T0–T30, T0–T60, or T30–T60 due to the induction of septic shock. The data is presented as mean ± one standard deviation.

The temporal variance in the measurements of the cohort was analysed between T0–T30, as well as T0–T60 and T30–T60 using paired sample *t*-tests. Temporal variance was analysed over T0–T60 and T30–T60 because sometimes the symptoms of septic shock do not manifest immediately after the endotoxin infusion at T30. Statistically significant changes (*P* < 0.05) were seen in the measured systolic and diastolic aortic and pulmonary artery pressures and right ventricular end diastolic volume over the first hour, showing the expected influence of the endotoxin infusion on the circulation.  Figure 4 shows these main hemodynamic measurements across the four animals.

### 3.1. Subject-Specific Model Validation

For all the subject-specific models, the model outputs matched the measurements used in the identification process (mean aortic and pulmonary pressures, aortic and pulmonary artery pulse pressure, and stroke volume) to percentage errors of less than 0.5%. This result is expected and indicates the convergence of the identification process.

**Figure 5:**
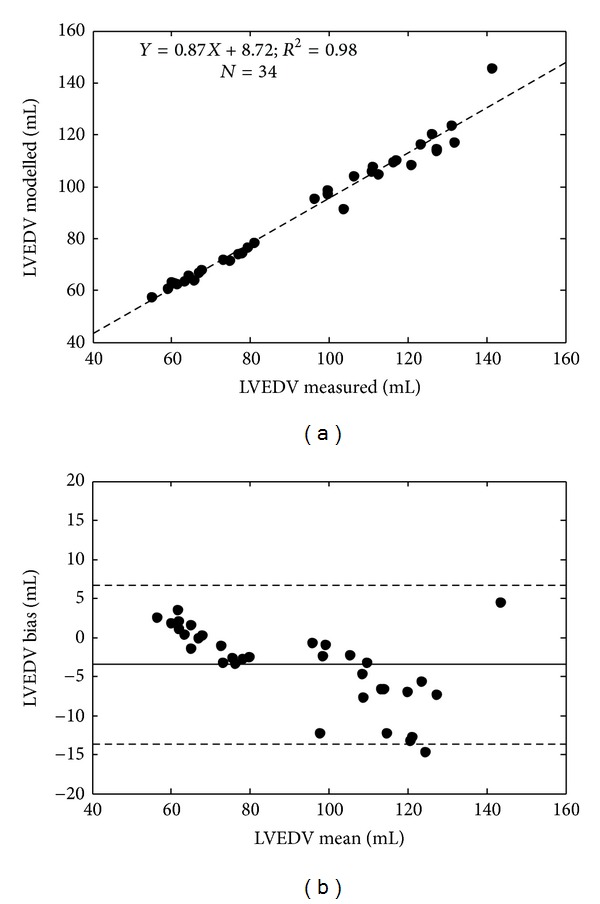
Regression (a) and Bland-Altman analysis showing 2 standard deviation limits (b) of the modelled and measured left ventricular end diastolic volume (LVEDV).

**Figure 6:**
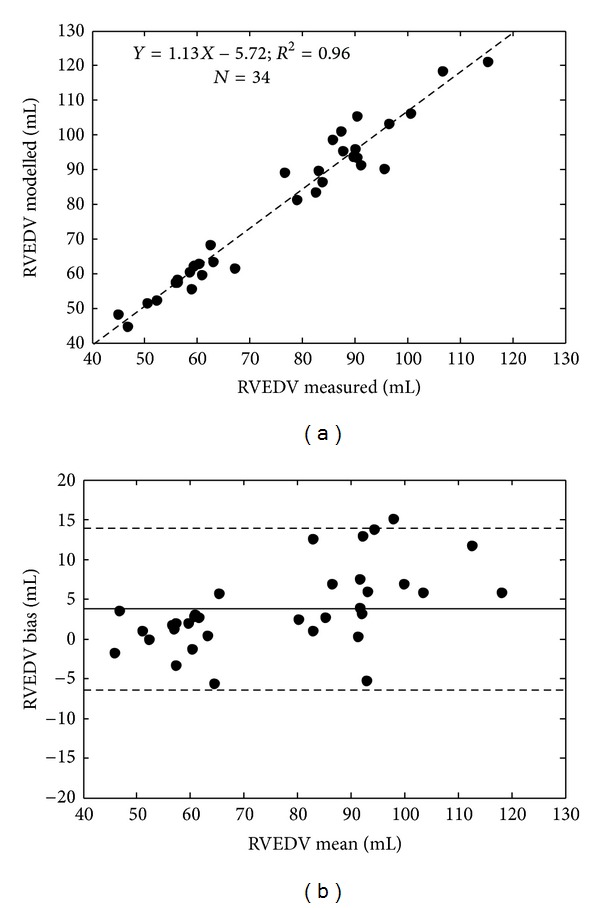
Regression (a) and Bland-Altman analysis showing 2 standard deviation limits (b) of the modelled and measured right ventricular end diastolic volume (RVEDV).

Validation was achieved by comparing model outputs of left and right ventricular end diastolic volumes (LVEDV, RVEDV) and maximum left and right ventricular pressures (*P*_lv,max⁡_, *P*_rv,max⁡_) to their corresponding measured value. These measurements were not used directly in the identification process, and thus, represent an independent validation of the method. Bias, precision, and correlation metrics were analysed for validation (Table 2 and Figures 5 and 6 for LVEDV and RVEDV). All four model outputs correlated well with the measured data (*R* ^2^ ≥ 0.76). Modelled left ventricular outputs (LVEDV, *P*_lv,max⁡_) had a small negative bias (−3.4 mL, −1.6 mmHg), whereas right ventricular outputs (RVEDV, *P*_rv,max⁡_) tended to slightly overestimate (3.8 mL, 4.5 mmHg) the true measurement. The precision (2 standard deviations) of the LVEDV and RVEDV predictions was 10.2 mL and 10.2 mL and 12.8 mmHg and 12.9 mmHg for *P*_lv,max⁡_ and *P*_rv,max⁡_. All percentage errors were within and acceptable range (<20%), except for the 95th percentile error in the modelled maximum right ventricular pressure. 

**Figure 7:**
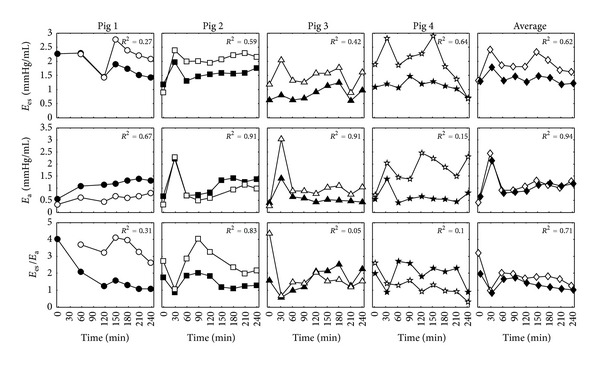
Comparison of subject-specific and averaged modelled right ventricle end systolic elastance (*E*_es, rvf_), pulmonary elastance (*E*_*a*_), and right ventricular arterial coupling (*E*_es,rvf_/*E*_*a*_), represented by the black symbols, against experimentally derived metrics from [23], represented by the white symbols.

For further validation, the temporal trends of the identified right ventricular end systolic elastance, pulmonary afterload, and right ventricular arterial coupling (RVAC), from the model, were compared to corresponding metrics (*E*_es,rvf_, *E*_*a*_, and *E*_es,rvf_/*E*_*a*_). These metrics were determined experimentally in [23] using right ventricular pressure-volume analysis, for *E*_es,rvf_, and a windkessel model, for *E*_*a*_. The afterload on the right ventricle in the model is the sum of the resistance of the pulmonary valve and pulmonary vasculature divided by the period of one heartbeat (*E*_*a*,model_ = [*R*_pv_ + *R*_pul_]/*T*). From Figure 7 it is seen that the subject-specific models in most cases track the experimental trends in *E*_es,rvf_ and *E*_*a*_. Weaker relationships are seen between the modelled and experimentally RVAC metrics (*E*_es,rvf_ /*E*_*a*_). However, the model did capture all the averaged temporal trends for the cohort with *R* ^2^ = 0.62, 94, and 0.71 for *E*_es,rvf_, *E*_*a*_, and *E*_es,rvf_ /*E*_*a*_. Note that experimental data was not available for Pig 1 at T0, T30, and T90, and Pig 2 at T150, due to difficulties performing the vena cava occlusion manoeuvre.

### 3.2. Septic Shock Trends

**Figure 8:**
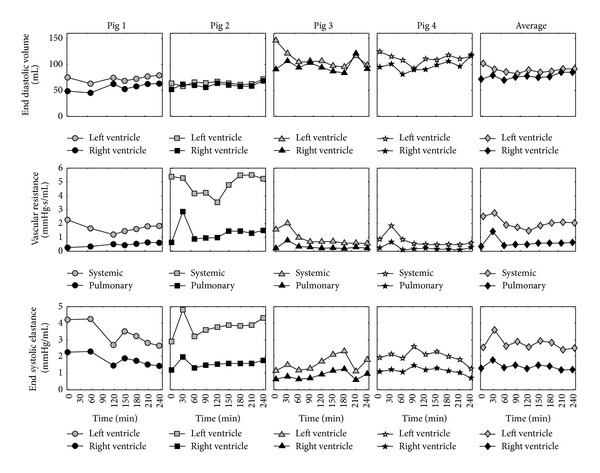
Modelled subject-specific indices of preload (end diastolic volume), afterload (vascular resistance), and contractility (end systolic elastance). The grey symbols represent the left ventricle or systemic metrics. The black symbols represent right ventricle or pulmonary metrics.

Averaged model metrics were used to test whether the subject-specific CVS models could capture the general trends of septic shock across the cohort of pigs. These metrics include ventricular preload, afterload, and contractility, three of the main determinants of cardiac and circulatory function. The averaged temporal trends for the metrics used in this analysis are shown in Figure 8. Please note that the spike in some of these metrics at T30 due to initial reaction of the pigs to endotoxin insult is not discussed in the following sections. The analysis of preload, afterload, and contractility, as presented in the following, only focuses on overall trends seen in these parameters over the four-hour duration of the trials. 

### 3.3. Preload

In the model, preload on the ventricles is represented by end diastolic volumes in the left and right ventricles (LVEDV, RVEDV). The average modelled LVEDV and RVEDV are shown in Figure 8. These results show the subject-specific models identified an increase in RVEDV during the study indicating the onset of right ventricle distension. In contrast, an initial decrease in LVEDV was observed, suggesting a decrease in venous return to the left heart immediately after the endotoxin infusion. However, LVEDV does start to increase during the second half of the trial indicating the activation of left ventricle compensatory mechanism which combats the effects of low venous return to the heart. Both these observations are known consequences of septic shock [25, 26].

### 3.4. Afterload

Another main hemodynamic consequence of septic shock is sudden decrease in left ventricle afterload due to a systemic inflammation [24–26]. In the model, the parameter systemic vascular resistance (*R*_sys_) represents the main component of the afterload on the left ventricle. The average trends for *R*_sys_, along with its pulmonary counterpart, pulmonary vascular resistance (*R*_pul_), can be seen in Figure 8. These identified trends show a significant decrease in *R*_sys_ after T60, symptomatic of sepsis. Moreover, a steady increase in *R*_pul_ after T60 was observed by the subject-specific CVS models, indicating an increase in right ventricle afterload due to the effects of the endotoxin. Again, both these findings are indicative of septic shock [24–26].

### 3.5. Contractility

In the subject-specific CVS models contractility is represented by parameters of left and right ventricular end systolic elastance (*E*_es,lvf_, *E*_es,rvf_), as shown in Figure 8. In septic shock a decrease in these parameters is expected due to myocardial depression [25, 26, 52, 53]. However, contrary to this expected trend, the averaged values for *E*_es,lvf_ and *E*_es,rvf_ stayed relatively constant over the trial. This unexpected outcome may be the consequence of the hemofiltration therapy, which has been shown to improve cardiac function during endotoxic shock in several experimental studies [23, 54, 55]. On the other hand, these results could be due to autonomous reflex responses, such as baroreflex control, attempting to maintain blood pressure by increasing contractility [56]. As a result, such mechanisms could be counteracting the effects of decreased myocardial state in the pigs. Since autonomous control mechanisms are not directly simulated in the CVS model it is impossible to fully distinguish if changes in the model parameters are due to the effects of these mechanisms or a result of the administered therapy.

### 3.6. Subject-Specific Response

Although the averaged results, seen previously, show that the model is capable of identifying the hemodynamic trends of the induced disease, it is also important to know how each subject responds to the disorder and corresponding treatment. Hence, it is essential to analyse the individual time varying trends for each pig, not just the averaged trends of the disease. Figure 8 illustrates the subject-specific response of *R*_sys_ to the endotoxic shock and LPHF therapy. On average, systemic resistance drops by 20.3% across the four pigs over the duration of the trials. However, when analysing the individual response of each of the pigs, it is seen that the *R*_sys_ returns to baseline for Pig 2, and a substantial improvement is observed in Pig 1 after LPHF treatment (T60 onwards). Whereas, there are large drops in *R*_sys_ with no apparent improvement with treatment in Pigs 3 and 4, indicating the difference response to septic shock between the pigs.

## 4. Discussion

### 4.1. Modelling Errors

For validation the model was compared to independent measurements that were not used in the identification process, as well as pulmonary hemodynamic indices calculated using an independent method [23]. Almost all ventricular volumes (LVEDV and RVEDV) and maximum pressures (*P*_lv,max⁡_ and *P*_rv,max⁡_) were identified to an error of less than 20% (except the 95th percentile error in the maximum right ventricular pressure), which is acceptable for this type of physiological monitoring. Furthermore, the time varying trends in these four measurements were identified to a high degree of accuracy, with calculated *R* ^2^ > 0.90, except for the maximum right ventricular pressure where *R* ^2^ = 0.76, which is still a very good result. These results suggest that subject-specific models of the CVS could be used to accurately monitor disease-dependent changes in ventricular preload in an intensive care environment. 

In most cases, the temporal trends of right ventricular end systolic elastance and pulmonary afterload from the subject-specific CVS models correlated well to experimentally derived measurements of *E*_es,rvf_ and *E*_*a*_ from [23]. The modelled results tended to underestimate *E*_es,rvf_. The primary reason for this bias is because of the assumption in the model identification process that the dead space volume of the right ventricle (*V*_*d*,rvf_) is constant and equal to 23 mL, as reported in [36]. In reality, *V*_*d*,rvf_ most likely changes as the contractile state of the pigs changes during the trials, but due to the lack of available measurements it cannot be identified. Hence, in the model the changes in contractile state are solely represented by *E*_es,rvf_. Poorer correlations were also noticed for RVAC. The reason for these poor relationships is most likely due to accumulation of errors in calculating this compound metric (*E*_es,rvf_/*E*_*a*_). These include the addition of error due to measurement noise in the experimental metrics and the addition of modelling errors in the identified parameters. However, a strong temporal relationship for RVAC could be seen of *R* ^2^ = 0.71 when the effects of measurement noise and modelling error were averaged over the cohort (as seen in Figure 7).

Noticeably, there were larger errors in the modelled *E*_es,rvf_ and *E*_*a*_ for Pig 4, as seen in Figure 7. This pig had a substantially higher heart rate than the other pigs, on average around 150 beats per minute compared to 122, 49, and 113 beats per minute for Pigs 1, 2, and 3. As a result, Pig 4 had a very high cardiac output up to seven times higher than the other pigs. In this extreme case, the CVS model was unable to capture the high flow dynamics of Pig 4. However, work is currently underway to improve the model for these types of cases.

A primary cause of some of the larger errors and difference in polarity of the mean errors between the left and right ventricle measurements, as seen in Table 2, is the inherent difficulty of measuring and calculating the right ventricular volume experimentally with a conductance catheter. Due to the complex shape of the right ventricle, the volume measurements were underestimated, especially for Pigs 3 and 4, where the left ventricular SV appeared to be more than double the right ventricular SV. The CVS is essentially a closed system and the ventricles pump in series. Hence, it is unsustainable for their SV of the ventricles to be a largely different for more than a couple of heartbeats, during a transient period, or else there would be a large buildup of volume somewhere in the circulation. However, the measurements were taken when the pigs were hemodynamically stable and should reflect steady state conditions. Hence, it is obvious that the measured right ventricular volume is underestimating the true volume in the right ventricle.

To overcome these measurement problems, the average value of the measured left and right ventricular stoke volumes was used in the model identification process for both ventricles. Thus, once converged, the identified models generally underestimated the measured left ventricular stoke volume and overestimated the measured right ventricular stroke volume. This issue caused the LVEDV and, consequentially, the left ventricular pressure in the model to be underestimated relatives to the measurements taken in the experiment, with the opposite occurring in the right ventricle. However, more importantly, these errors were generally systematic, so the trends associated with these measurements are still clearly identified and accurate.

### 4.2. Detecting Septic Shock

The subject-specific models of the CVS captured the main hemodynamic trends of septic shock. A drop in systemic vascular resistance and an increase in pulmonary vascular resistance were identified. As a result, an increase in RVEDV was also seen in the subject-specific CVS models. These changes are well-known consequences of the disease [24–26]. Hence, these results indicate that the identified models are able to accurately capture the expected trends of septic shock in the pigs.

### 4.3. Subject-Specific Modelling

The results show that personalised CVS models can be used to accurately match and predict important hemodynamic markers of afterload (*R*_sys_, *R*_pul_), preload (LVEDV, RVEDV), and contractility (*E*_es,lvf_, *E*_es,rvf_). Clinically, the contractile state and preload on the ventricles can be extremely difficult to continuously measure using traditional techniques. Generally, LVEDV and RVEDV can only be estimated intermittently using echocardiography, and there exists no widely accepted clinical measure of contractility. The model-based approach, used in this paper, provides a way to continuously estimate preload and contractility. These model-based metrics could be used to help guide therapy such as using fluids to increase LVEDV and the administering inotropes to control *E*_es,lvf_ and *E*_es,rvf_. The accurate and continuous monitoring of preload is especially important in septic shock, as it has been shown that early goal-directed fluid resuscitation can increase patient outcomes [57]. Hence, metrics derived from identified subject-specific CVS models could help provide more meaningful targets for similar goal-directed therapies.

The subject-specific models were also able to track the main hemodynamic changes resulting from induced septic shock. These results show that the new model identification method utilised is capable of accurately identifying markers of cardiovascular health, like the previous integral-based method [19, 20] but requires a much smaller, more clinically available set of measurements including features (mean, amplitude, and maximum gradient) of the aortic and pulmonary artery pressures, SV, GEDV, and the mitral and tricuspid valve closure times. This measurement set is significantly smaller than the one used in the integral-based approach, in the sense that only discrete measurements are used instead of full pressure and volume waveforms. However, this new measurement set represents the most important features of these waveforms. For example, instead of fitting the integral of the aortic pressure the new method fits the mean, amplitude, and maximum gradient of aortic pressure in the CVS models, which are of more clinical importance. Hence, although less data is used to identify subject-specific CVS models, the models are actually matched to larger number of features within this data. Therefore, the smaller measurement set does not decrease the number of parameters that can be identified in these models, and its use increases the clinical applicability of the parameter identification method.

From the subject-specific CVS models, identified from this minimal set of measurements, the contrasting reaction of the pigs in response to the induced disease could be seen. Initially, *R*_sys_ decreased in all the pigs, but in later stages of the trials *R*_sys_ increased in Pigs 1 and 2, increasing aortic blood pressure in these pigs. These results tentatively suggest that the LPHF was responsible for increasing *R*_sys_ through the removal inflammatory inducing molecules from the blood stream. However, this treatment appeared to have little effect on Pigs 3 and 4. Larger trials would be required before a more substantiated conclusion on this therapy could be made, which was not the goal of this research. However, it is clear that the identified CVS models were able to accurately segregate those subjects who did (and did not) respond positively to the endotoxin insult, due either to autonomous compensatory reflexes or because of the effects of this therapy.

The parameter identification method used in this study is a significant improvement on previous work [19, 20] especially with respect to clinical feasibility. Although the measurements in this study were only taken intermittently every 30 minutes, the system has the potential to provide continuous real-time information to medical staff. Accurate model identification can be achieved using low fidelity pressure measurements, as only the main features of the aortic and pulmonary artery pressure are required such as the mean, amplitude, and maximum gradient. In practice, the aortic pressure could be inferred from the radial artery pressure using several possible methods [42, 44, 46, 47]. The method could also be implemented in the ICU at little extra cost, as it only uses measurements already taken in the ICU. Thus, the approach could be applied without adding further invasive burden to the patient. In addition, the system can be easily automated, so that a full picture of a patient's hemodynamic state is available to clinicians with little extra effort required from medical staff.

### 4.4. Limitations

The number of parameters that can be identified in the CVS model is limited by the paucity of available measurements in the ICU, and thus, the amount of measurements that were used to identify pig-specific CVS models in this research. In this study only 14 out of a possible 37 parameters of the CVS model were identified. However, these 14 parameters control all the important dynamics in the model, including the contractility of the ventricles, vascular stiffness, and resistance to flow through parts of the CVS. In a clinical setting, these parameters can be controlled using common therapies including the administration of inotropes, vasoactive drugs, and/or fluid resuscitation. The other 23 parameters primarily have lesser effects, such as the inertia of blood through the heart valves, or are relatively constant over the population and are thus modelled as such, enabling accurate relative trend identification. The good validation and diagnostic results obtained in this study support the choice of model parameters to be identified.

A further limitation is the use of systemic and vascular resistance as the primary metric for ventricular afterload, although this approach is widely used [58–61]. A more complicated description of right ventricular afterload was utilised for validation, as shown earlier in Figure 7, so that the magnitude and units of the metric aligned with the experimentally derived value. However, clinically, systemic and pulmonary vascular resistances are still the most commonly used definitions of afterload [58]. Hence, the systemic and pulmonary vascular resistances were chosen for examination of afterload in this study.

## 5. Conclusion

This study presented a method for identifying pig-specific CVS models of endotoxic shock. These models were able to identify expected disease-dependent changes in ventricular preload, afterload, and contractility in the pigs, using typically available ICU measurements. These indices of cardiac function can be difficult, impossible, or impractical to monitor clinically but are extremely useful to know when guiding cardiovascular therapy. Hence, this model-based approach could potentially be used to help monitor these determinants of cardiac function and assist clinicians with diagnosis and treatment-based decisions.

## Figures and Tables

**Figure 9:**
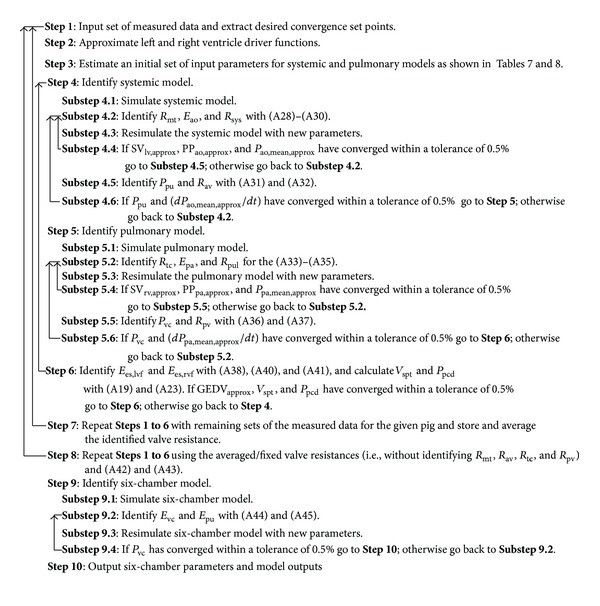
Step-by-step overview of the parameter identification used to create subject-specific CVS models.

**Table 1:** List of identified parameters and corresponding measurements used to identify them for the simplified systemic, simplified pulmonary, and six-chamber models. Each line on the table represents a measurement/parameter pair, where the measurement on the line is used to directly identify the corresponding parameter. All the parameters in this table, except for the valve resistances, are uniquely identified for each set of measurements. The valve resistances are identified for each set of measurements and then averaged for a subject.

Model	Measurement		Identified parameter	
	Name	Symbol	Name	Symbol
Systemic	Global end diastolic volume	GEDV	Left ventricular elastance	*E*_es,lvf_
	Stroke volume	SV	Mitral valve resistance	*R*_mt_
	Mean aortic pressure	MAP	Systemic vascular resistance	*R*_sys_
	Aortic pulse pressure	PP_ao_	Aortic elastance	*E*_ao_
	Maximum ascending aortic gradient	*dP*_ao,max_/*dt*	Aortic valve resistance	*R*_av_
	Mitral valve closure time	*t*_mt_	Pulmonary vein pressure	*P*_pu_

Pulmonary	Global end diastolic volume	GEDV	Right ventricular elastance	*E*_es,rvf_
	Stroke volume	SV	Tricuspid valve resistance	*R*_tc_
	Mean pulmonary artery pressure	MPAP	Pulmonary valve resistance	*R*_pul_
	Pulmonary artery pulse pressure	PP_pa_	Pulmonary artery elastance	*E*_pa_
	Maximum ascending pulmonary artery pressure	*dP*_pa,max_/*dt*	Pulmonary valve resistance	*R*_pv_
	Tricuspid valve closure time	*t*_tc_	Vena cava pressure	*P*_vc_

6 chamber	Vena cava pressure (identified from systemic model)	*P*_vc_	Vena cava elastance	*E*_vc_
	Pulmonary vein pressure (identified from pulmonary model)	*P*_pu_	Pulmonary vein elastance	*E*_pu_

**Table 2:** The median and range of the measured left and right end diastolic volumes (LVEDV, RVEDV) and maximum left and right ventricular pressures (*P*_lv,max_, *P*_rv,max_). Bias and precision metrics (2SD), median percentage errors with 5th and 95th percentile bounds, and correlation coefficients of the modelled LVEDV, RVEDV, *P*_lv,max_, and *P*_rv,max_.

	Median (range)	Bias ± 2SD	% Error (5th–95th percentile)	*R* ^2^
LVEDV	97.7 mL (55.0–141.2)	−3.4 ± 10.2 mL	3.5% (0.5–10.9)	0.98
RVEDV	93.3 mL (44.8–138.2)	3.8 ± 10.2 mL	4.9% (0.4–16.3)	0.96
*P*_lv,max_	86.1 mm Hg (63.2–121.8)	−1.6 ± 12.8 mm Hg	3.8% (0.4–16.4)	0.96
*P*_rv,max_	38.7 mm Hg (18.0–60.7)	4.5 ± 12.9 mm Hg	14.8% (2.6–29.9)	0.76

**Table 3:** Identified CVS model parameters and the measurements (convergence set points) used to identify them.

Identified parameter		Measurement used to identify parameter	
Symbol	Description	Symbol	Description
*E*_es,lvf_	Left ventricle end systolic elastance	GEDV	Global end diastolic volume
*E*_ao_	Aortic elastance	PP_ao_	Amplitude of aortic pressure
*E*_vc_	Vena cava elastance	*P*_vc_	Modelled Vena cava pressure
*E*_es,rvf_	Right ventricle end systolic elastance	GEDV	Global end diastolic volume
*E*_pa_	Pulmonary artery elastance	PP_ao_	Amplitude of pulmonary artery pressure
*E*_pu_	Pulmonary vein elastance	*P*_vc_	Modelled vena cava pressure
*R*_mt_	Mitral valve resistance	SV	Stroke volume
*R*_av_	Aortic valve resistance	*dP*_ao,max_/*dt*	Maximum ascending aortic pressure gradient
*R*_sys_	Systemic vascular resistance	*P*_ao,mean_	Mean aortic pressure
*R*_tc_	Tricuspid resistance	SV	Stroke volume
*R*_pv_	Pulmonary valve resistance	*dP*_ao,max_/*dt*	Maximum ascending pulmonary artery pressure gradient
*R*_pul_	Pulmonary vascular resistance	*P*_po,mean_	Mean pulmonary artery pressure
*P*_vc_	Vena cava pressure	*t*_tc_, SV	Tricuspid valve closure time and stroke volume
*P*_pu_	Pulmonary vein pressure	*t*_mt_, SV	Mitral valve closure time and stroke volume

**Table 4:** Identified cardiac driver functions (normalised time varying elastances) and the measurements used to calculate them.

Identified cardiac activation		Measurement used to identify waveform	
Symbol	Description	Symbol	Description
driL(*t*)	Left ventricle normalised time varying elastance	*P*_ao_, GEDV	Aortic pressure waveform and global end diastolic volume
driR(*t*)	Right ventricle normalised time varying elastance	*P*_pa_, GEDV	Pulmonary artery pressure and global end diastolic volume

**Table 5:** CVS model constants (from [28, 29]).

Symbol	Description	Value
*P*_0,lvf_	Parameter of left ventricle EDPVR	0
*λ*_lvf_	Parameter of left ventricle EDPVR	0.033
*V*_*d*,lvf_	Parameter of left ventricle ESPVR	23
*P*_0,rvf_	Parameter of right ventricle EDPVR	0
*λ*_rvf_	Parameter of right ventricle EDPVR	0.023
*V*_*d*,rvf_	Parameter of right ventricle ESPVR	23
*V*_*d*,spt_	Parameter of septal ESPVR	2
*V*_0,spt_	Parameter of septal EDPVR	2
*λ*_spt_	Parameter of septal EDPVR	0.435
*P*_0,spt_	Parameter of septal EDPVR	1.1101
*E*_es,spt_	Septum elastance	48.7540
*P*_0,pcd_	Parameter of pericardium	0.5003
*V*_0,pcd_	Parameter of pericardium	200
*λ*_pcd_	Parameter of pericardium	0.03
*P*_th_	Intrathoracic pressure	0
*V*_*d*,ao_	Aortic unstressed volume	0
*V*_*d*,vc_	Vena cava unstressed volume	0
*V*_*d*,pa_	Pulmonary artery unstressed volume	0
*V*_*d*,pu_	Pulmonary vein unstressed volume	0
*L*_mt_	Mitral valve inertance	0
*L*_av_	Aortic valve inertance	0
*L*_tc_	Tricuspid valve inertance	0
*L*_pv_	Pulmonary valve inertance	0

**Table 6:** Full list of the CVS model outputs. Check marks indicate outputs that are also experimentally measured.

Symbol	Description	Measured
*V*_lv_	Left ventricle volume	√
*V*_ao_	Aorta volume	
*V*_vc_	Vena cava volume	
*V*_rv_	Right ventricle volume	√
*V*_pa_	Pulmonary artery volume	
*V*_pu_	Pulmonary vein volume	
*P*_lv_	Left ventricle pressure	√
*P*_ao_	Aorta pressure	√
*P*_vc_	Vena cava pressure	
*P*_rv_	Right ventricle pressure	√
*P*_pa_	Pulmonary artery pressure	√
*P*_pu_	Pulmonary vein pressure	
*Q*_mt_	Mitral valve flow rate	
*Q*_av_	Aortic valve flow rate	
*Q*_sys_	Systemic flow rate	
*Q*_tc_	Tricuspid valve flow rate	
*Q*_pv_	Pulmonary valve flow rate	
*Q*_pul_	Pulmonary flow rate	
*V*_spt_	Septum volume	
*P*_pcd_	Pericardium pressure	

**Table 7:** Initial systemic model parameter inputs.

Parameter	*P*_pu_	*R*_mt_	*E*_es,lvf_	*R*_av_	*E*_ao_	*R*_sys_	*P*_vc_
Initial value	5	0.05	2	0.04	2.5	2.5	5

**Table 8:** Initial pulmonary model parameter inputs.

Parameter	*P*_vc_	*R*_tc_	*E*_es,rvf_	*R*_pv_	*E*_pa_	*R*_pul_	*P*_pu_
Initial value	5	0.04	0.8	0.03	2.1	0.4	5

## Appendix

The CVS model is defined by (A.1)–(A.16). A full list of the model parameters, constants, and outputs is listed in Tables 3, 4, 5, and 6. Equation (A.1) describe the flow, *Q*, through the heart valves. These first-order differential equations state that the pressure (*P*) difference across a valve is proportional to the sum of the resistive (*QR*) and inertial effects $\left(L\dot{Q}\right)$ on the flow as follows:

(A.1) $$\begin{array}{c} L_{\text{av}}{\dot{Q}}_{\text{av}}=P_{\text{lv}}-P_{\text{ao}}-Q_{\text{av}}R_{\text{av}},\\ L_{\text{mt}}{\dot{Q}}_{\text{mt}}=P_{\text{pu}}-P_{\text{lv}}-Q_{\text{mt}}R_{\text{mt}},\\ L_{\text{pv}}{\dot{Q}}_{\text{pv}}=P_{\text{rv}}-P_{\text{pa}}-Q_{\text{pv}}R_{\text{pv}},\\ L_{\text{tc}}{\dot{Q}}_{\text{tc}}=P_{\text{vc}}-P_{\text{rv}}-Q_{\text{tc}}R_{\text{tc}}. \end{array}$$

Nonvalvular flow is represented using Ohm's law (A.2). Hence, the flow through the vasculature is proportional to the pressure difference across the section divided by the resistance to flow as follows:

(A.2) $$\begin{array}{c} Q_{\text{sys}}=\frac{P_{\text{ao}}-P_{\text{vc}}}{R_{\text{sys}}},\\ Q_{\text{pul}}=\frac{P_{\text{pa}}-P_{\text{pu}}}{R_{\text{pul}}}. \end{array}$$

Pressure in the noncardiac chambers is proportional to the elastance (*E*) and stressed blood volume within the chamber. In these chambers the elastances are assumed to be constant over a heartbeat. The stressed blood volume in (A.3) is represented as the total blood volume (*V*) minus the unstressed volume (*V*_*d*_). Chambers within the thoracic cavity, as shown in Figure 1, are additionally influenced by the intrathoracic pressure (*P*_th_) in the thoracic cavity as follows:

(A.3) $$\begin{array}{c} P_{\text{pu}}=E_{\text{pu}}\left(V_{\text{pu}}-V_{d,\text{pu}}\right)+P_{\text{th}},\\ P_{\text{pa}}=E_{\text{pa}}\left(V_{\text{pa}}-V_{d,\text{pa}}\right)+P_{\text{th}},\\ P_{\text{vc}}=E_{\text{vc}}\left(V_{\text{vc}}-V_{d,\text{vc}}\right)+P_{\text{th}},\\ P_{\text{ao}}=E_{\text{ao}}\left(V_{\text{ao}}-V_{d,\text{ao}}\right). \end{array}$$

The free wall pressure of the left and right ventricles (*P*_lvf_, *P*_rvf_) is calculated using (A.4). These equations ignore the effects of the intraventricular septum and pericardium which are taken into account later with (A.11)–(A.19), hence, the name free wall pressures. Myocardial activation of the left and right ventricles are represented using normalised time varying elastance curves (driL and driR) which vary in time between 0 and 1. The first term in both of these equations represent the percentage of the pressure generation in the ventricle due to dynamic contraction of the heart muscles. At maximum contraction, driL and driR equals 1, and the pressure volume relationship in the ventricles is represented by their end systolic pressure volume relationship (ESPVR). The second term represents the percentage of pressure generation due to the passive elastic recoil of the ventricular chambers. When driL and driR equal 0, the pressures in these chambers are bound by their end diastolic pressure volume relationship (EDPVR). In between end systole and end diastole the nonlinear ventricular pressure volume relationship is defined as a weighted sum of the ESPVR and EDPVR. In other words, (A.4) represents the percentage of dynamic and passive pressure generation that is occurring in the ventricles at any point in time, as defined by

(A.4) Plvf=driL·Ees,lvf·(Vlvf−Vd,lvf)+(1−driL)·P0,lvf·(eλlvf(Vlvf − V0,lvf)−1),Prvf=driR·Ees,rvf·(Vrvf  −  Vd,rvf)+(1−driR)·P0,rvf·(eλrvf(Vrvf  −  V0,rvf)−1).

The volume of blood in the noncardiac chambers varies at a rate proportional to the flow exiting and entering those chambers, as described by (A.5)–(A.8). Furthermore, (A.9) and (A.10), representing the volume in the left and right ventricles, take into consideration the nonlinear effects of valvular flow by using Heaviside functions. The use of Heaviside functions in these equations ensures that there is no backwards flow through the heart valves as follows:

(A.5) $$\begin{array}{c} {\dot{V}}_{\text{pv}}=Q_{\text{pul}}-Q_{\text{mt}}, \end{array}$$

(A.6) $$\begin{array}{c} {\dot{V}}_{\text{pa}}=Q_{\text{pv}}-Q_{\text{pul}}, \end{array}$$

(A.7) $$\begin{array}{c} {\dot{V}}_{\text{vc}}=Q_{\text{sys}}-Q_{\text{tc}}, \end{array}$$

(A.8) $$\begin{array}{c} {\dot{V}}_{\text{ao}}=Q_{\text{av}}-Q_{\text{sys}}, \end{array}$$

(A.9) $$\begin{array}{c} {\dot{V}}_{\text{rv}}=\text{Heaviside}\left(Q_{\text{tc}}\right)Q_{\text{tc}}-\text{Heaviside}\left(Q_{\text{pv}}\right)Q_{\text{pv}}, \end{array}$$

(A.10) $$\begin{array}{c} {\dot{V}}_{\text{lv}}=\text{Heaviside}\left(Q_{\text{mt}}\right)Q_{\text{mt}}-\text{Heaviside}\left(Q_{\text{av}}\right)Q_{\text{av}}. \end{array}$$

Finally, ventricular interaction is modelled through considering the action of the intraventricular septum and the pericardium on cardiac dynamics. Equation (A.11) represents all the pressures acting on the intraventricular septum, where the muscle activation of the septum (driS) is assumed to be equal to the average of the left and right ventricular normalised time varying elastance curves (driL, driR). From (A.11) the blood volume displaced by the septum, *V*_spt_, can be evaluated and used to calculate the real left and right ventricular volumes (*V*_lv_, *V*_rv_), as shown by (A.13) and (A.14) as follows:

(A.11) driS·Ees,spt·(Vspt−Vd,spt)+(1−driS)   ·P0,spt·(eλspt(Vspt   −   V0,spt)−1)  =driL·Ees,lvf·(Vlv−Vspt)+(1−driL)   ·P0,lvf·(eλlvf(Vlv   −   Vspt)−1)−driR·Ees,rvf   ·(Vrv+Vspt)−(1−driR)·P0,rvf   ·(eλrvf(Vrv   +   Vspt)−1),

(A.12) $$\begin{array}{c} \text{driS}=\frac{\text{driL}+\text{driR}}{2}, \end{array}$$

(A.13) $$\begin{array}{c} V_{\text{lvf}}=V_{\text{lv}}-V_{\text{spt}}, \end{array}$$

(A.14) $$\begin{array}{c} V_{\text{rvf}}=V_{\text{rv}}+V_{\text{spt}}. \end{array}$$

The pressure generated by the pericardium (*P*_pcd_), which encases the heart, is proportional to the sum of the left and right ventricular volumes (*V*_pcd_) as described by (A.15) and (A.16). The total external pressure acting on the cardiac chamber (*P*_peri_), as seen in (A.17)–(A.19), is therefore the sum of the pericardium and intrathoracic pressure, as the heart is located in the thoracic cavity. Please see [28, 30–32], for a more detailed description of the derivation of the CVS model equations. One has

(A.15) $$\begin{array}{c} P_{\text{pcd}}=P_{0,\text{pcd}}\left(e^{\lambda_{\text{pcd}}(V_{\text{pcd}}\;\;-\;\;V_{0,\text{pcd}})}-1\right), \end{array}$$

(A.16) $$\begin{array}{c} V_{\text{pcd}}=V_{\text{lv}}+V_{\text{rv}}, \end{array}$$

(A.17) $$\begin{array}{c} P_{\text{peri}}=P_{\text{pcd}}+P_{\text{th}}, \end{array}$$

(A.18) $$\begin{array}{c} P_{\text{lv}}=P_{\text{lvf}}+P_{\text{peri}}, \end{array}$$

(A.19) $$\begin{array}{c} P_{\text{rv}}=P_{\text{rvf}}+P_{\text{peri}}. \end{array}$$

Equations (A.17)–(A.34) make up the identification process.  Table 3 lists all the parameters identified in the identification process and convergence set points used to identify them.  Table 4 lists the driver functions, also known as time varying elastances used to simulate the effect of cardiac contraction in the left and right ventricles in the CVS model, and Tables 7 and 8 list the initial parameters estimates used to commence the parameter identification process.

Initially, the driver functions for the left and right ventricles are estimated using GEDV and features from the aortic pressure waveform (for driL) and pulmonary artery pressure waveform (for driR) using the method described in [34, 35]. The systemic model is identified first. The model is initially simulated using the parameters values given in Table 7. A method is then used to iteratively simulate and improve the estimates of *R*_mt_, *E*_ao_, and *R*_sys_ in the systemic submodel by matching the modelled SV, PP_ao_ and *P*_ao,mean_ to their measured value as follows:

(A.20) $$\begin{array}{c} R_{\text{mt},\text{new}}=\left(\frac{{\text{SV}}_{\text{lv},\text{approx}}}{{\text{SV}}_{\text{true}}}\right)R_{\text{mt},\text{old}}, \end{array}$$

where SV_lv,approx_ = max⁡(*V*_lv_) − min⁡(*V*_lv_),

(A.21) $$\begin{array}{c} E_{\text{ao},\text{new}}=\left(\frac{{\text{PP}}_{\text{ao},\text{true}}}{{\text{PP}}_{\text{ao},\text{approx}}}\right)E_{\text{ao},\text{old}}, \end{array}$$

where PP_ao_ = max⁡(*P*_ao_) − min⁡(*P*_ao_),

(A.22) $$\begin{array}{c} R_{\text{sys},\text{new}}=\left(\frac{P_{\text{ao},\text{mean},\text{true}}}{P_{\text{ao},\text{mean},\text{approx}}}\right)R_{\text{sys},\text{old}}, \end{array}$$

where *P*_ao,mean_ = (max⁡(*P*_ao_) + min⁡(*P*_ao_))/2.

Once the modelled SV, PP_ao_, and *P*_ao,mean_ have converged, new estimates of *P*_pu_ and *R*_av_ are identified. *R*_mt_, *E*_ao_, and *R*_sys_ are then reidentified with the new estimates of *P*_pu_ and *R*_av_, and the whole process is repeated until *P*_pu_ does not change between iterations, and *dP*_ao,max⁡_/*dt* converges to its measured value. In this process, *P*_pu_ and *R*_av_ are identified independently of *R*_mt_, *E*_ao_, and *R*_sys_, as they interact and are dependent on the correct convergence of these parameters as follows:

(A.23) $$\begin{array}{c} P_{\text{pu}}=P_{\text{lv}}\left(t_{\text{mt}}\right), \end{array}$$

(A.24) $$\begin{array}{c} R_{\text{av},\text{new}}=\frac{\left(dP_{\text{ao},\max,\text{approx}}/dt\right)}{\left(dP_{\text{ao},\max,\text{true}}/dt\right)R_{\text{av},\text{old}}}. \end{array}$$

During this process *E*_es,lvf_ is left as its constant value to be identified later in the identification process.

The same approach is used to identify the pulmonary sub-model, as the pulmonary model essentially mirrors the systemic model. *R*_tc_, *E*_pa_, and *R*_pul_ are identified first. Then *P*_vc_ and *R*_pv_ are updated, these values are used to reidentify *R*_tc_, *E*_pa_, and *R*_pul_, and the process is repeated until SV, PP, *P*_ao,mean_, *P*_vc_, and *dP*_ao,max⁡_/*dt* have converged. During this process *E*_es,rvf_ is held as its original value to be identified later. Consider

(A.25) $$\begin{array}{c} R_{\text{tc},\text{new}}=\left(\frac{{\text{SV}}_{\text{rv},\text{approx}}}{{\text{SV}}_{\text{true}}}\right)R_{\text{tc},\text{old}}, \end{array}$$

where SV_rv,approx_ = max⁡(*V*_rv_) − min⁡(*V*_rv_),

(A.26) $$\begin{array}{c} E_{\text{pa},\text{new}}=\left(\frac{{\text{PP}}_{\text{pa},\text{true}}}{{\text{PP}}_{\text{pa},\text{approx}}}\right)E_{\text{pa},\text{old}}, \end{array}$$

where PP_pa_ = max⁡(*P*_pa_) − min⁡(*P*_pa_),

(A.27) $$\begin{array}{c} R_{\text{pul},\text{new}}=\left(\frac{P_{\text{pa},\text{mean},\text{true}}}{P_{\text{pa},\text{mean},\text{approx}}}\right)R_{\text{pul},\text{old}}. \end{array}$$

where *P*_pa,mean_ = (max⁡(*P*_pa_) + min⁡(*P*_pa_))/2

(A.28) $$\begin{array}{c} P_{\text{vc}}=P_{\text{rv}}\left(t_{\text{tc}}\right) \end{array}$$

(A.29) $$\begin{array}{c} R_{\text{pv},\text{new}}=\frac{\left({dP}_{\text{pa},\max,\text{approx}}/dt\right)}{\left({dP}_{\text{pa},\max,\text{true}}/dt\right)R_{\text{pv},\text{old}}} \end{array}$$

Once, the systemic and pulmonary submodelss have converged, *E*_es,lvf_ and *E*_es,rvf_ are updated. First, the sum of the end systolic elastances (*E*_es,sum_) is updated by matching GEDV as follows:

(A.30) $$\begin{array}{c} E_{\text{es},\text{sum},\text{new}}=\frac{{\text{GEDV}}_{\text{approx}}}{{\text{GEDV}}_{\text{true}}}E_{\text{es},\text{sum},\text{old}}, \end{array}$$

where GEDV_approx_ = max⁡(V_lv_) + max⁡(V_rv _).

Then a relationship, derived from the afterloads on the left and right ventricles, is used to calculate the relative contribution of *E*_es,lvf_ from this sum. The derivation of this relationship is shown in [22] as follows:

(A.31) CE=Pao,mean,true−PvcPao,mean,true+Ppa,mean,true≈Ees,lvfEes,lvf+Ees,rvf=const,

(A.32) $$\begin{array}{c} E_{\text{es},\text{lvf}}=C_{E,\text{mean}}E_{\text{es},\text{sum}} \end{array}$$

Finally, *E*_es,rvf_ can also be found using

(A.33) $$\begin{array}{c} E_{\text{es},\text{rvf}}=E_{\text{es},\text{sum},\text{new}}-E_{\text{es},\text{lvf}}. \end{array}$$

During this step in the identification process, ventricular interaction, in the form of septal and pericardial dynamics (*V*_spt_ and *P*_pcd_) are calculated and added to the systemic and pulmonary submodelss to couple the systems. Once *E*_es,lvf_ and *E*_es,rvf_ have been updated, and *V*_spt_ and *P*_pcd_ have been calculated, the systemic and pulmonary models are reidentified using the new values for these parameters and interactions. This overall process, for identifying *E*_es,lvf_ and *E*_es,rvf_, is repeated until GEDV converges, thus completing the identification of the systemic and pulmonary models.

The systemic and pulmonary models are then identified for the remaining measurement sets, T0–T240 for that pig. The valve resistance are averaged across these models with these averaged valve resistance used to reidentify the systemic and pulmonary models. Hence, (A.20), (A.24), (A.25), and (A.29) are no longer required, and (A.23) and (A.28) are replaced by (A.34) and (A.35) for identifying *P*_pa_ and *P*_vc_ as follows:

(A.34) $$\begin{array}{c} P_{\text{pu},\text{new}}=\frac{{\text{SV}}_{\text{lv},\text{approx}}}{{\text{SV}}_{\text{true}}}P_{\text{pu},\text{old}}, \end{array}$$

(A.35) $$\begin{array}{c} P_{\text{vc},\text{new}}=\frac{{\text{SV}}_{\text{rv},\text{approx}}}{{\text{SV}}_{\text{true}}}P_{\text{vc},\text{new}}. \end{array}$$

Once the systemic and pulmonary models have been identified with the averaged valve resistances, the final parameters of the six-chamber model, *E*_vc_ and *E*_pu_, can be identified. This is done by using the parameters of systemic and pulmonary to simulate the six-chamber CVS model. The six-chamber model is iteratively simulated, and (A.36) and (A.37) are used to identify *E*_vc_ and *E*_pu_. The parameter identification process is concluded when the mean of the six-chamber *P*_vc,6_ and *P*_pu,6_ matches their corresponding identified values from the simplified submodels (*P*_vc,simple_, *P*_pu,simple_), producing a full six-chamber subject-specific model of the CVS as follows:

(A.36) $$\begin{array}{c} E_{\text{vc},\text{new}}=\left(\frac{P_{\text{vc},\text{simple}}}{\text{mean}\left(P_{\text{vc},6}\right)}\right)E_{\text{vc},\text{old}}, \end{array}$$

(A.37) $$\begin{array}{c} E_{\text{pu},\text{new}}=\frac{P_{\text{pu},\text{simple}}}{\text{mean}\left(V_{\text{pu}}\right)}. \end{array}$$

Figure 9 outlines the steps used to identify the parameters of the six-chamber CVS model.
